# A Systematic Comparative Assessment of the Response of Ovarian Cancer Cells to the Chemotherapeutic Cisplatin in 3D Models of Various Structural and Biochemical Configurations—Does One Model Type Fit All?

**DOI:** 10.3390/cancers14051274

**Published:** 2022-03-01

**Authors:** Priyanka Gupta, Aline Miller, Adedamola Olayanju, Thumuluru Kavitha Madhuri, Eirini Velliou

**Affiliations:** 1 Centre for 3D Models of Health and Disease, Division of Surgery and Interventional Science, University College London, London W1W 7TY, UK; priyanka.g.gupta@ucl.ac.uk; 2 Bioprocess and Biochemical Engineering Group (BioProChem), Department of Chemical and Process Engineering, University of Surrey, Surrey GU2 7XH, UK; 3 Manchester BIOGEL, 19F4, Mereside, Alderley Park, Alderley Edge, Chesire SK10 4TG, UK; a.miller@manchesterbiogel.com (A.M.); a.olayanju@manchesterbiogel.com (A.O.); 4 Department of Gynaecological Oncology Royal Surrey NHS Foundation Trust, Egerton Road, Guildford GU2 7XX, UK; docmadhuri231@gmail.com; 5 Honorary Senior Lecturer in Cancer Research, School of Applied Sciences, University of Brighton, Huxley Building, Lewes Road, Brighton BN2 4GJ, UK

**Keywords:** epithelial ovarian cancer, tissue engineering, 3D in vitro model, chemotherapy, Cisplatin, spheroids, hydrogels, polymeric scaffolds, A2780, SK-OV-3

## Abstract

**Simple Summary:**

Epithelial Ovarian Cancer is considered to be a ‘silent killer’ and a challenge for gynaecological health across the world due to its asymptotic nature in the early stages, its late-stage diagnosis, high recurrence rate and resistance to currently available treatment methods (chemotherapy). These disheartening figures highlight the need for extensive in vitro studies to better understand this disease. A number of in vitro 3D models are currently available to aid in the study of ovarian cancer and its response to therapeutic methods. In this work, we report, for the first time, a comprehensive comparative study of three widely used 3D in vitro models for ovarian cancer, along with chemotherapy assessment of primary and metastatic cells. Our study highlights the importance of selecting an appropriate 3D in vitro platform, which is based on multiple factors including the origin of cells used, experimental time period and experimental design, even for one specific disease.

**Abstract:**

Epithelial Ovarian Cancer (EOC) is a silent, deadly and aggressive gynaecological disease with a relatively low survival rate. This has been attributed, to some extent, to EOC’s high recurrence rate and resistance to currently available platinum-based chemotherapeutic treatment methods. Multiple groups have studied and reported the effect of chemotherapeutic agents on various EOC 3D in vitro models. However, there are very few studies wherein a direct comparative study has been carried out between the different in vitro 3D models of EOC and the effect of chemotherapy within them. Herein, we report, for the first time, a direct comprehensive systematic comparative study of three different 3D in vitro platforms, namely (i) spheroids, (ii) synthetic PeptiGels/hydrogels of various chemical configurations and (iii) polymeric scaffolds with coatings of various extracellular matrices (ECMs) on the cell growth and response to the chemotherapeutic (Cisplatin) for ovary-derived (A2780) and metastatic (SK-OV-3) EOC cell lines. We report that all three 3D models are able to support the growth of EOC, but for different time periods (varying from 7 days to 4 weeks). We have also reported that chemoresistance to Cisplatin, in vitro, observed especially for metastatic EOC cells, is platform-dependent, in terms of both the structural and biochemical composition of the model/platform. Our study highlights the importance of selecting an appropriate 3D platform for in vitro tumour model development. We have demonstrated that the selection of the best platform for producing in vitro tumour models depends on the cancer/cell type, the experimental time period and the application for which the model is intended.

## 1. Introduction

Epithelial Ovarian Cancer (EOC) is the seventh most common cancer amongst women across the world and the third most common gynaecological cancer, ranked after cervical and uterine [1]. Even with extensive advancements in the field of cancer diagnosis and treatment, the 5-year survival rate of ovarian cancer is only around 30% [2] and it is the most common cause of gynaecological cancer-related deaths worldwide [3,4]. The relatively high mortality rate of ovarian cancer is attributed to its asymptotic nature, delayed onset and recognition of symptoms, lack of proper screening, high recurrence rate along with resistance to available chemotherapeutic methods of treatment [5,6]. The current gold standard for EOC treatment involves ‘debulking’ via reductive surgery in combination with the use of platinum-based chemotherapy involving chemotherapeutic agents such as Cisplatin and Carboplatin [7,8,9]. However, it has been observed that over 80% of patients have a relapse post chemotherapy, along with the development of a platinum-resistant aggressive form of EOC, all of which can be attributed, to a large extent, to EOC’s complex tumour microenvironment (TME) [10,11,12,13]. The latter is a cocktail of different cellular, structural, biochemical (extracellular matrix (ECM) protein composition), biophysical and biomechanical features, all of which interact in complex and sometimes unknown ways with the tumour, leading to its progression, resistance to treatment and metastasis. Overall, these disheartening data suggest that there is an unmet need for ex vivo models of EOC in order to better understand the disease, EOC’s complex TME and its unique mode of metastasis, as well as to predict patient-specific, personalised drug responses.

Similar to other cancers, traditionally, EOC studies including treatment screening are carried out either in (i) 2D in vitro systems such as T-Flasks and petri dishes [14,15,16,17,18] or (ii) in vivo, in animal models such as mice, rats and hens [19,20,21]. Firstly, 2D tumour models are easy to use, reproducible and are generally responsive to most therapeutic methods [22,23,24]. However, they are unable to capture key properties of the in vivo TME, including cell–cell interactions, cell–ECM interactions, structure, stiffness, spatial orientation and various environmental gradients [25,26] that are typically formed in a 3D tumour during growth and progression. In vivo animal models are currently considered to be the gold standard for therapeutic assessment, as they are more realistic in terms of capturing the in vivo organ complexity in comparison to 2D systems [27,28,29,30]. However, these models are expensive, difficult to reproduce and time-consuming. Additionally, there is evidence suggesting that they undergo genetic changes that differ from the evolutionary course of human diseases, raising further concern regarding their validity as models for personalised treatment [31,32,33,34].

Thus, 3D in vitro models are slowly emerging to tide over these issues associated with 2D as well as animal models. To date, 3D models used for ovarian cancer in vitro research include (i) cell spheroids, (ii) hydrogels and (iii) natural or synthetic biomaterial-based polymeric scaffolds. Spheroids are the most commonly used 3D in vitro models for ovarian cancer [24,35,36,37,38,39,40,41,42]. The earliest known spheroid model of EOC was reported in 1995 by Griffon et al. [43], wherein they developed cellular aggregates/spheroids from patient samples and exposed them to photon radiation (0–8 Gy), followed by analysis after 7 days post-treatment. They reported that the effect of radiation on the spheroids was dependent on the spheroids’ size and proposed that spheroids are able to mimic the patient-specific radio-response to a large extent [43]. Since then, many groups have reported the feasibility of using EOC spheroids developed via different methods of fabrication, as suitable 3D models for obtaining an understanding of EOC and for predicting therapeutic outcomes in vitro [35,36,37,38,40,41,44,45,46,47,48,49]. In general, these therapeutic assessment studies report observing higher chemoresistance in spheroid models in comparison to 2D monolayer models for different established cell lines as well as patient samples. For example, recently, Gunay et al. (2020), in their comparative study between OVCAR-3 and OVCAR-8 cell lines, reported that different cell lines have different responses to Taxol and Cisplatin [44]. Similarly, Raghavan et al. (2017) reported that different patient samples responded differently to the chemotherapeutic treatment in spheroid-based 3D models, highlighting the feasibility of using the model for the personalised screening of therapy [35]. An extensive comparison of 3D spheroid models using 16 different commercially available EOC cell lines was carried out by Heredia-Soto et al. (2018). Similarly to other groups, they reported that 3D models for the different cell lines showed higher resistance to Cisplatin treatment in comparison to 2D monolayer models [38]. The study also reported that, for most cell lines, there was increased expression of master EMT regulators in 3D models in comparison to 2D, showing the advantage of 3D models in capturing the biochemical 3D features of the EOC TME. 

Although simple cell-based spheroids are proven to be feasible models for therapeutic assessment and very promising tools especially for fast drug screening, they come with certain inherent constraints, including limited culture time; the formation of unrealistically high gradients of nutrients and oxygen, which can lead to the formation of extreme/extended necrotic cores at the spheroid centre; a lack of robust ECM mimicry and a lack of spatial architecture and structural orientation [26,32,50,51]. However, 3D models based on hydrogels and polymeric scaffolds can solve some of the issues associated with simple cell-based spheroid models. More specifically, they can sustain a longer culture period (several weeks), they enable better diffusion of biochemical reagents and they allow for the presence of specific ECM proteins, spatial orientation and a relatively defined and tuneable architecture [50,51]. Several groups have developed and used both natural and synthetic material-based hydrogels as 3D models of EOC for the purpose of therapeutic assessment [52,53,54,55,56,57,58,59,60,61,62]. For example, Yang and Zhao (2011) carried out a comparative study between a collagen I hydrogel and a RADA16-I peptide hydrogel with different cell lines (A2780, A2780/DDP and SK-OV-3) using three different chemotherapeutic agents (5-FU, Paclitaxel and curcumin). They reported that synthetic hydrogels with RADA16-I peptide were able to maintain all three cell lines in culture and, similarly to spheroids, hydrogel-based 3D models of EOC showed approximately 2- to 5-fold higher chemoresistance in comparison to 2D monoculture [53]. Liu et al. (2018) reported the growth of patient-derived platinum-sensitive and platinum-resistant cell lines within a collagen I hydrogel, for 7 days, wherein they observed the overexpression of mesenchymal markers (N-cadherin, vimentin and fibronectin) and transcriptional factors (snail and slug) along with higher chemoresistance in comparison to 2D monolayer systems [61]. Chen et al. (2014) used a commercially available Basement Membrane Extract (BME) hydrogel to compare the characteristics of a CD44^+^/CD117^+^ double-positive EOC cancer stem cell (CSC) population between 3D (the hydrogel) and a 2D culture system [59]. They attributed the increased chemoresistance of the CSC population in the 3D hydrogel to the increased expression of ABCG2, ABCB1, MMP-2 and MMP-9 as compared to 2D. The longest study for EOC using hydrogels as 3D models was conducted by Loessner et al. (2010), wherein EOC cell lines OV-MZ-6 and SK-OV-3 were cultured in PEG hydrogels biofunctionalised with RGD, Gln and MMP-sensitive sites for 14 days, followed by treatment using Paclitaxel and 7-day post-treatment analysis [58].

Currently, there are very few reported studies wherein polymeric scaffolds have been used for the development of ovarian cancer 3D models [63,64,65,66]. However, to the best of our knowledge, there are currently no reported studies wherein polymeric scaffold-based 3D models of EOC have been used for therapeutic assessment. Girard et al. (2013) developed a nanofibrous polymeric scaffold composed of PLGA and mPEG-PLA polymers (3P scaffold), via electrospinning, to culture BG-1 ovarian cancer cell lines for up to 5 days, wherein the EOC cells were shown to be growing as cell aggregates [63]. Ul-Islam et al. (2019) developed a 3D model of EOC using A-2780 ovarian cancer cell lines and a chitosan and bacterial cellulose-based polymeric scaffold and maintained it for 7 days [65]. 

In addition to these static monocellular models of EOC (containing cancer cells only), efforts have also been made to study the effect of shear stress and fluid flow, as well as the presence of other stromal cells, e.g., mesothelial cells and adipocytes, in EOC 3D models [62,66,67,68,69,70,71,72,73]. 

Despite extensive advancement in the field of EOC in vitro 3D models, there are very few publications available for a direct comparative study between the various types of in vitro 3D models, i.e., simple cell spheroids vs. hydrogels vs. polymeric scaffolds, and most of them compare either different types of hydrogels or spheroid models prepared via different fabrication methods [52,74]. For example, Zheng et al. (2014), carried out a comparative study between hydrogels from collagen I, Matrigel, alginate and agarose using SK-OV-3 cell lines, injected in vivo in a nude mice model for a period of 4 weeks [52]. They reported that tumour formation by SK-OV-3 cells was best supported by collagen, followed by Matrigel, alginate, control (cell suspension only) and agarose in vivo, along with increased MMP activity and upregulated expression of laminin, fibronectin, HIF-1α and VEGF-A in collagen I hydrogels. They concluded that the bioactive and biomimetic hydrogels were superior to ‘inert’ (i.e., lacking in native ligands that allow mammalian cells to attach) hydrogels at promoting tumour regeneration/growth. A comparative study between different fabrication methods for spheroid formation (polydimethylsiloxane-based microfluidic chips, ultra-low-attachment plates and hanging drop method) and their effect on drug sensitivity for carboplatin was carried out by Patra et al. (2020), wherein they highlighted the challenges of choosing appropriate preclinical models for drug testing [74]. 

In this current study, we have taken a step further to systematically compare the chemotherapeutic (Cisplatin) response (cell viability and apoptosis analysis) between spheroid, hydrogel and polymeric scaffold-based 3D in vitro models of epithelial ovarian cancer. For such a comparative study, we have used two different cell lines, namely (i) A2780, which is derived from the ovary (primary tumour), and (ii) SK-OV-3, which is derived from the ascites fluid (metastatic site), to identify the impact of cell origin on the growth and drug response in various 3D systems.

## 2. Materials and Methods

### 2.1. Cell Culture

Human Epithelial Ovarian Cancer (EOC) cell lines A2780 (Merck, Gillingham, UK) and SK-OV-3 (HTB 77, ATCC, Teddington, UK) were cultured in RPMI-1640 (Thermo Fisher Scientific, Loughborough, UK) and McCoy’s 5a (Thermo Fisher Scientific, Loughborough, UK) media, respectively, supplemented with 10% Foetal Bovine Serum (FBS, Thermo Fisher Scientific, Loughborough, UK), 2 mM glutamine (Merck, Gillingham, UK) and 1% antibiotic–antimycotic (Thermo Fisher Scientific, Loughborough, UK) in a humidified incubator at 37 °C and 5% CO_2_. Both cell lines were passaged regularly upon reaching 75–80% confluency with Typle E (Thermo Fisher Scientific, Loughborough, UK) till the required cell densities were obtained. The two cell lines were selected to reflect two different stages of ovarian cancer. A 2780 cell line was derived from the ovarian tumour of an untreated patient while SK-OV-3 was derived from the ascites fluid post-metastasis.

### 2.2. 3D Cell Culture and Chemotherapeutic Treatment on Polymeric Scaffolds

Polyurethane (PU) polymeric scaffolds were prepared via the Thermal-Induced Phase Separation (TIPS) method, sterilised and coated (surface modified with physisorption) with the ECM proteins fibronectin and collagen I, as previously described [75,76,77]. Both proteins are important elements of the EOC TME [78,79]. The scaffolds were highly porous (85% porosity) and they were microporous, with interconnected pores with an average pore diameter of 100–150 μm. A2780 and SK-OV-3 cell lines were seeded in the scaffolds (5 × 5 × 5 mm^3^) at a seeding density of 0.5 × 10^6^ cells/scaffold and cultured for 28 days. Post 28 days (4 weeks) of culture, the chemotherapeutic agent Cisplatin (Merck, Gillingham, UK) was added to the culture at a concentration of 50 µM for 1 feeding cycle (48 h) and removed thereafter. The concentration of Cisplatin was selected based on published IC_50_ data for A2780 and SK-OV-3 in 3D spheroids [38]. The scaffolds were then characterised 24 h post-treatment with sectioning, staining, microscopy and image analysis. 

### 2.3. 3D Cell Culture and Chemotherapeutic Treatment on Synthetic Hydrogels

Synthetic PeptiGels (Manchester BIOGEL, Manchester, UK) were also used for our study with A2780 and SK-OV-3 cells. An initial fast screening was carried out between 4 different hydrogels of different stiffness and charge: α1 (5 kPa, neutral charge), α2 (10 kPa, medium charge,), α3 (5 kPa, low charge) and α4 (1 kPa, high charge) [60,80]. Based on this preliminary screening (data not shown), the α4 PeptiGel was selected for further long-term studies as it led to optimal cell proliferation and longer hydrogel chemical stability. In order to incorporate ECM mimicry, α4 hydrogel conjugated with RGD (cell binding sequence found in fibronectin) and GFOGER (integrin binding site withing collagen I) were also used. The incorporation of these ECM protein conjugates enabled a more biomimetic synthetic system and also allowed us to compare the hydrogels with our protein-coated PU scaffolds (see Section 2.4 below). PeptiGels were used as per manufacturer’s instructions. Briefly, both A2780 and SK-OV-3 cells were encapsulated via physical mixing of 100 μL of cell suspension into 1 mL of hydrogel, providing a final cell concentration of 0.5 × 10^6^ cells/mL. Thereafter, cells were mixed to ensure a homogeneous solution. Aliquots of 200 μL of hydrogels with cells were pipetted into 24-well cell culture inserts with 1 mL of cell culture media added to each well and 200 μL on top of each gel within the inserts. Thereafter, the cell culture plates were incubated at 37 °C and 5%CO_2_. Cell culture medium was changed every 20 min for the first hour to calibrate the hydrogel to neutral pH and every 2 days thereafter and cultured for a period of 3 weeks. Post 3 weeks of culture, the chemotherapeutic agent Cisplatin (Merck, Gillingham, UK) was added to the culture at a concentration of 50µM for 1 feeding cycle (48 h) and removed thereafter. Hydrogels were then assessed 24 h post-treatment via staining, microscopy and image analysis (see following sections).

### 2.4. 3D Cell Culture and Chemotherapeutic Treatment on Cellular Spheroids

Spheroids of A2780 and SK-OV-3 cell lines were fabricated using specialised 96-well round-bottom plates, provided by faCellitate (Manheim, Germany). More specifically, to fabricate the spheroids, 200 µL of cell suspension containing 25,000 cells was seeded in each well and cultured for 1 week with media change every 2 days. Post 1 week of culture, chemotherapeutic agent Cisplatin (Merck, Gillingham, UK) was added to the culture at a concentration of 50 µM for 1 feeding cycle (48 h) and removed thereafter. Spheroids were then assessed 24 h post-treatment via staining, microscopy and image analysis (Cytation 5, BioTek, Agilent Technologies, Stockport, UK). 

### 2.5. Spatial Evaluation of Live and Dead Cells via Imaging

To visualise the spatial distribution of live and dead cells pre- and post-treatment, model-specific methods were used. More specifically, PU scaffolds were collected at appropriate time points, snap-frozen in liquid nitrogen for 15 min and then preserved at −80 °C for further analysis, as previously described [75,76,77]. This method has been widely used in the field of tissue engineering for sample preservation without harming the cells [81,82,83]. Prior to analysis, scaffolds were sectioned and washed twice with PBS. For live/dead cell analysis, a Live/Dead Viability/Cytotoxicity Kit was used (Molecular Probes, Thermo Scientific, Loughborough, UK). Scaffold sections were stained with 2 μM of Calcein-AM (4 mM stock) and 4 μM of ethidium homodimer (2 mM stock) and were then incubated at 37 °C for 1 h. The solution was then removed, and the samples were washed twice in PBS, followed by imaging using a Nikon Ti-Eclipse inverted confocal microscope (Nikon Instruments, Surbiton, UK).

PeptiGels and spheroids were stained and imaged live at appropriate time points. Unlike the PU polymeric scaffolds, snap-freezing the spheroids and the hydrogels was not feasible as this process would have destroyed their native structure. Hydrogels and spheroids were stained with 2 μM of calcein-AM (4 mM stock) and 4 μM of ethidium homodimer (2 mM stock) and were incubated at 37 °C for 2 h for proper penetration of the dyes. The solution was then removed, and the samples were washed twice in PBS followed by imaging. PeptiGels were imaged whole using a Nikon Ti-Eclipse inverted confocal microscope (Nikon Instruments, Surbiton, UK). Spheroids were imaged using Cytation 5 Cell Imaging Reader (BioTek, Agilent Technologies, Stockport, UK).

### 2.6. Spatial Evaluation of Apoptotic Cells (Caspase 3/7 Activity) via Imaging

The caspase 3/7 activity was visualised and quantified in situ to assess the induction of cellular apoptosis after different treatments. As described in Section 2.5, model-specific methods were used to stain and image the different 3D models. Post processing the different 3D models similarly to Section 2.5, samples were incubated in culture medium containing (i) the Cell Event Caspase-3/7 green detection reagent (Fisher Scientific, Loughborough, UK) and (ii) DAPI, 1:200 (Fisher Scientific, Loughborough, UK) for 1 h (PU scaffolds) and 2 h (PeptiGels and spheroids) at 37 °C. The presence of caspase 3/7 positive cells (green) was immediately evaluated with a Nikon Ti-Eclipse inverted confocal microscope (Nikon Instruments, Surbiton, UK) for PU scaffolds and PeptiGels. Spheroids were imaged using Cytation 5 Cell Imaging Reader (Biotek, Agilent Technologies, Stockport, UK).

### 2.7. Advanced Microscopy Imaging

Immunofluorescent samples of PU scaffolds and PeptiGels (prepared as described in Section 2.5 and Section 2.6 above) were imaged on a Nikon Ti-Eclipse inverted confocal microscope (Nikon Instruments, Surbiton, UK) and processed with the NIS-Elements software, using 405, 488 and 561 nm lasers for DAPI (blue), green fluorescence (calcein and caspase 3/7) and ethidium homodimer (red) staining, respectively. Confocal images were captured using a 10× objective and a 5–10 μm Z-stack distance. The same acquisition conditions were used for the positive controls. Cytation 5 Cell imaging Reader (BioTek, Agilent Technologies, Stockport, UK) was used with similar lasers and 10× magnification with Z-stacking and montage creation to image the spheroid models, using 405, 488 and 561 nm lasers for DAPI (blue), green fluorescence (calcein and caspase 3/7) and ethidium homodimer (red) staining, respectively. Imaging was carried out using 10× objective along with Z-stack and the Montage feature of the instrument in order to image the complete spheroid construct. 

Multiple samples as well as multiple areas and multiple sections per sample were imaged for all models under study to ensure reproducibility. Representative images are presented in this manuscript.

### 2.8. Image Analysis and Quantification

Image-based quantification was carried out in a model-specific manner. Within PU scaffolds and PeptiGels, for the quantitative evaluation of (i) live (green) and dead (red) populations, as well as (ii) caspase-positive/apoptotic (green) and non-apoptotic (blue) populations of each image, the percentage of green vs. red (live/dead) or green vs. blue (caspase-positive/caspase-negative) areas of each image was calculated using Image J^®^ software (Wayne Rasband, NIH, Bethesda, MD, USA). The particle analyser macro (Image J^®^, Wayne Rasband, NIH, Bethesda, MD, USA) was used in each individual channel (green or read for live/dead and green or blue for caspase 3/7-DAPI, respectively). Multiple samples (*n* ≥ 3) were imaged and analysed for statistical relevance.

In the spheroid model, the average mean grey values for calcein (Live-Dead) and caspase 3/7 (apoptotic) were calculated using the particle measure macro in Image J^®^ software (Wayne Rasband, NIH, Bethesda, MD, USA). Multiple samples (*n* ≥ 3) were imaged and analysed for statistical relevance.

### 2.9. Statistical Analysis

Statistical analysis was performed for at least 3 independent experiments with at least 3 replicates per time point (*n* ≥ 3, *n* ≥ 3). Analysis of variance (one-way ANOVA) followed by the Bonferroni’s multiple comparison test or T-Test (depending on sample) using the GraphPad Prism^®^ software (version 8.00 for Windows) were carried out depending on samples, in order to find statistically significant differences between data (*p* < 0.05). Untreated samples were considered as controls in all cases. The error bars in the graphs represent the standard error of mean.

## 3. Results

### 3.1. Assessment of the Impact of the Chemotherapeutic Cisplatin on EOC Cells in a 3D Spheroid Model

**Figure 1:**
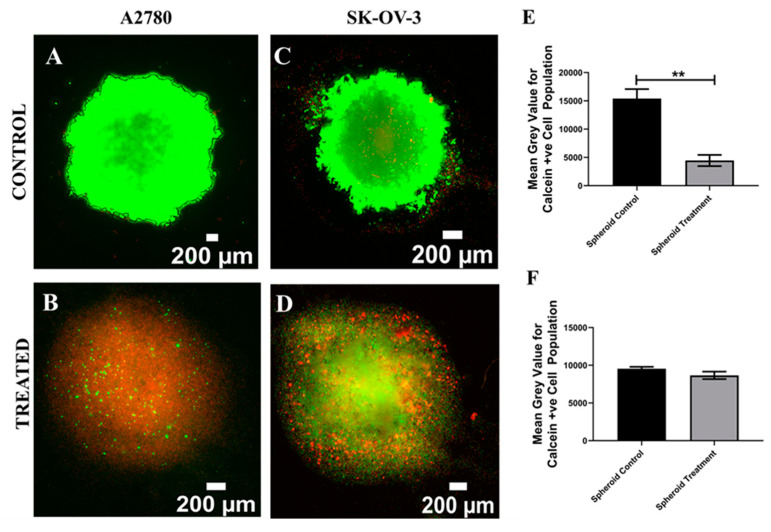
Effect of the chemotherapeutic Cisplatin on the viability EOC spheroids 24 h post-treatment (**A**–**D**): Representative images for live–dead (green–red) staining for untreated (control) and Cisplatin-treated A2780 and SK-OV-3 spheroids. (**E**) Image analysis-based quantification of live (green) image areas for A2780-treated and untreated spheroids. (**F**) Image analysis-based quantification of live (green) image areas for SK-OV-3-treated and untreated spheroids. Scale bar = 200 µm. Quantitative data represent mean ± SEM for multiple images (≥3) and multiple spheroids (≥3). ** *p* ≤ 0.01.

**Figure 2:**
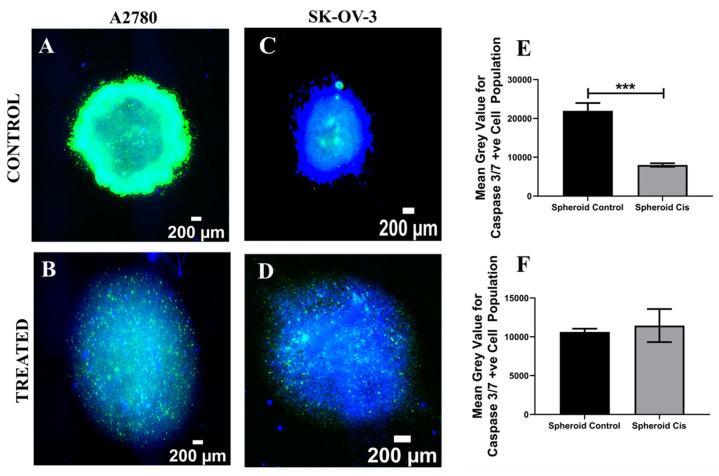
Effect of the chemotherapeutic Cisplatin on the apoptosis of EOC spheroids 24 h post-treatment (**A**–**D**): Representative images for caspase 3/7 (apoptosis)–DAPI (green–blue) staining for untreated (control) and Cisplatin-treated A2780 and SK-OV-3 spheroids. (**E**) Image analysis-based quantification of apoptotic (green) image areas for A2780-treated and untreated spheroids. (**F**) Image analysis-based quantification of apoptotic (green) image areas for SK-OV-3-treated and untreated spheroids. Scale bar = 200 µm. Quantitative data represent mean ± SEM for multiple images (≥3) and multiple spheroids (≥3). *** *p* ≤ 0.001.

As mentioned before, spheroids are the first in vitro 3D culture system established in tissue engineering and they have been extensively used for the therapeutic assessment of EOC [35,43,44]. In this study, Cisplatin, a widely used chemotherapeutic agent for EOC [35,44], was introduced to the culture medium for both A2780 and SK-OV-3 EOC spheroids on day 7 of culture [40,44] at a concentration of 50 µM for 48 h [38] (Section 2.4). Thereafter, Cisplatin-containing medium was replaced with fresh medium and the spheroids were maintained for 24 h, followed by post-treatment analysis of viability and apoptosis. More specifically, spheroid staining, imaging and image processing were carried out as described in Section 2.5, Section 2.6, Section 2.7 and Section 2.8, for spatial assessment and the quantification of the impact of Cisplatin on the cell viability and cell apoptosis for A2780 and SK-OV-3 spheroids (Figure 1 and Figure 2). A2780 spheroids were larger in size (2194 ± 130 µm) in comparison to SK-OV-3 spheroids (1305 ± 203 µm). Post treatment with Cisplatin, aggregate loosing was observed for both cell lines, with a resulting increase in spheroid mean diameter (A2780 = 2477 ± 168 µm; SK-OV-3 = 2093 ± 37 µm).

Figure 1A–D show representative images for live–dead staining for A2780 and SK-OV-3 spheroids, for both Cisplatin-treated and untreated (control) spheroids. Figure 1E,F show the equivalent quantification of the percentage of live areas from image-based analysis. 

As can be seen in Figure 1, 24 h post Cisplatin treatment, A2780 spheroids show a statistically significant decrease in cell viability as compared to untreated spheroids (Figure 1A,B,E). In contrast, no significant cell death was observed for SK-OV-3 cell lines 24 h post-treatment. This suggests that 50 µM of Cisplatin had an immediate and extremely damaging effect on A2780 spheroids, while its effect on SK-OV-3 viability in spheroids was much less, indicating some degree of resistance to Cisplatin.

In terms of apoptosis induction, we noticed a high amount of caspase 3/7 positive (apoptotic) cells present even in the untreated spheroids for both the cell lines, with A2780 control spheroids having a significantly higher apoptotic cell number in comparison to the Cisplatin-treated samples (Figure 2A,B,E). For SK-OV-3, both treated and untreated spheroids showed a high amount of apoptotic cells (Figure 2C,D,F). This suggests that both EOC cell lines had started undergoing programmed cell death post 10 days of culture in a spheroid-based culture, most likely due to diffusional and spatial limitations that are inherent to this 3D culture method. 

### 3.2. Assessment of the Impact of the Chemotherapeutic Cisplatin on EOC Cells in a Synthetic Peptide Hydrogel 3D Model

As mentioned in Section 2.3, peptide-based synthetic hydrogels were used for long-term culture (3 weeks) and Cisplatin chemotherapeutic assessment of A2780 and SK-OV-3 ovarian cancer cell lines. At the first stage, a preliminary screening was carried out to compare between hydrogels and different stiffness and charge combinations, and α4 PeptiGel was selected for long-term study as it was the hydrogel supporting the best viability and hydrogel stability for the timeframe of our experiments, i.e., 3 weeks (see also Section 2.3). As described in Section 2.3, in order to incorporate ECM mimicry, which has a crucial effect on cancer development and treatment response [84,85,86], ECM matrix-conjugated α4 PeptiGels were also tested. More specifically, RGD- and GFOGER-conjugated hydrogels were tested, mimicking fibronectin and collagen, respectively. Cisplatin was introduced to the culture medium for both A2780 and SK-OV-3 hydrogels at 3 weeks [58] of culture and at a concentration of 50 µM for 48 h [38] (Section 2.3). Thereafter, Cisplatin-containing medium was replaced with fresh medium and the hydrogels were maintained in culture for 24 h followed by post-treatment analysis.

**Figure 3:**
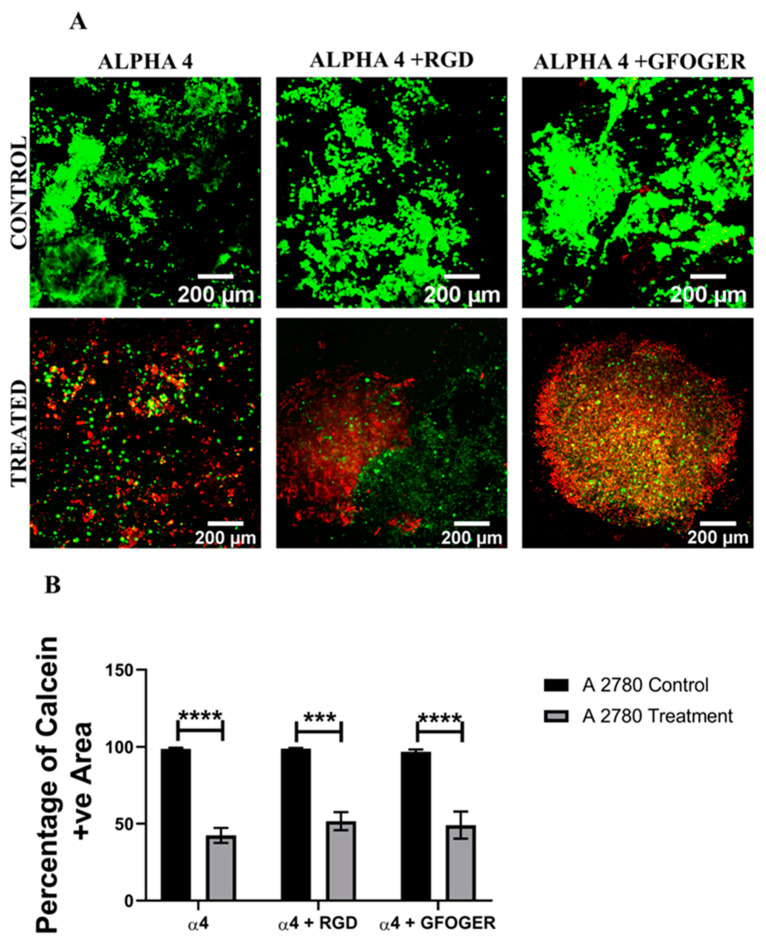
Effect of the chemotherapeutic Cisplatin on the viability of A2780 EOC cells grown in different synthetic PeptiGels, 24 h post-treatment (**A**): Representative images for live–dead (green–red) staining for both treated and untreated (control) A2780 PeptiGels. (**B**) Image analysis-based quantification of live (green) image areas for A2780 cells grown in the peptides. Scale bar = 200 µm. Quantitative data represent mean ± SEM for multiple images (≥3) and multiple hydrogels (≥3). *** *p* ≤ 0.001, **** *p* ≤ 0.0001.

**Figure 4:**
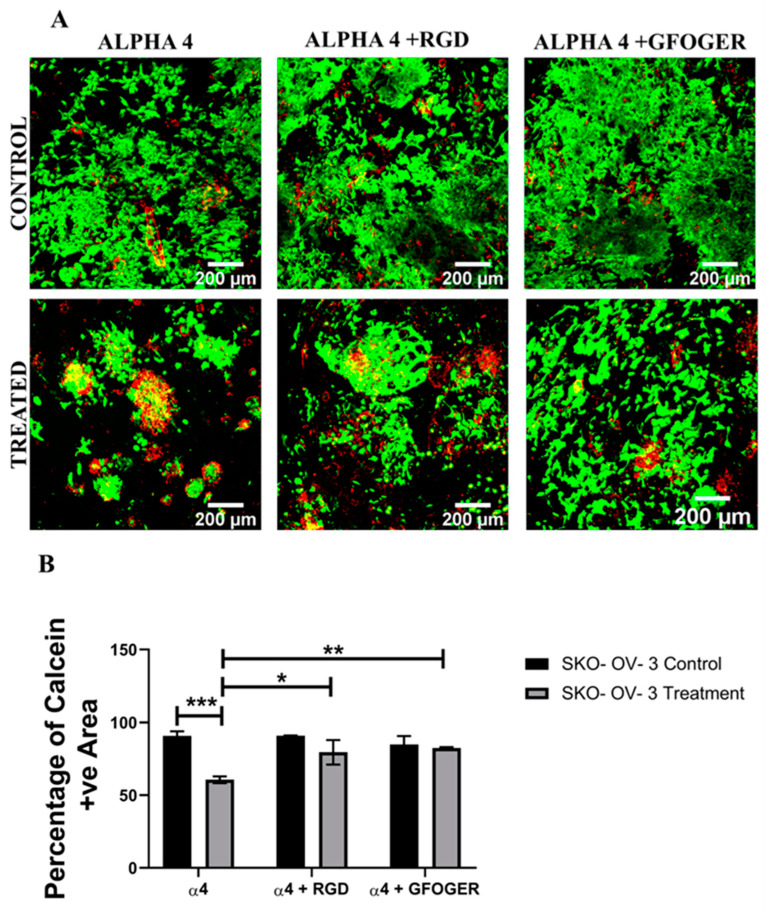
Effect of the chemotherapeutic Cisplatin on the viability of SK-OV-3 EOC cells grown in different PeptiGels, 24 h post-treatment (**A**): Representative images for live–dead (green–red) staining for both treated and untreated (control) SK-OV-3 PeptiGels. (**B**) Image analysis-based quantification of live (green) image areas for SK-OV-3 cells grown in the PeptiGels. Scale bar = 200 µm. Quantitative data represent mean ± SEM for multiple images (≥3) and multiple hydrogels (≥3). * *p* ≤ 0.05, ** *p* ≤ 0.01, *** *p* ≤ 0.001.

**Figure 5:**
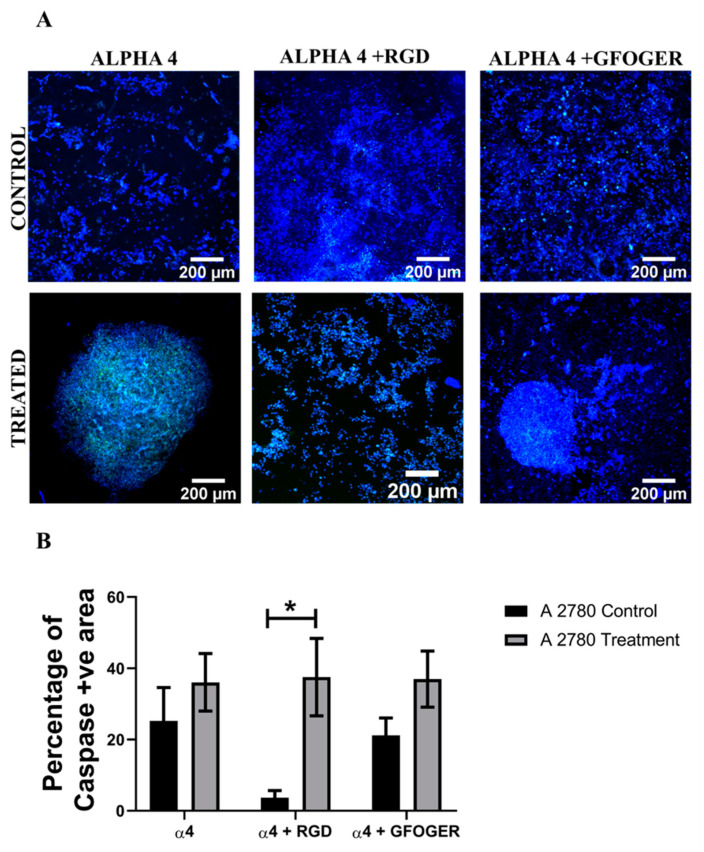
Effect of the chemotherapeutic Cisplatin on the apoptosis of A2780 EOC cells grown in different PeptiGels, 24 h post-treatment (**A**): Representative images for caspase 3/7 (apoptosis)–DAPI (green–blue) staining for both treated and untreated (control) A2780 PeptiGels. (**B**) Image analysis-based quantification of apoptotic (green) image areas for A2780 cells grown in the PeptiGels. Scale bar = 200 µm. Quantitative data represent mean ± SEM for multiple images (≥3) and multiple hydrogels (≥3). * *p* ≤ 0.05.

**Figure 6:**
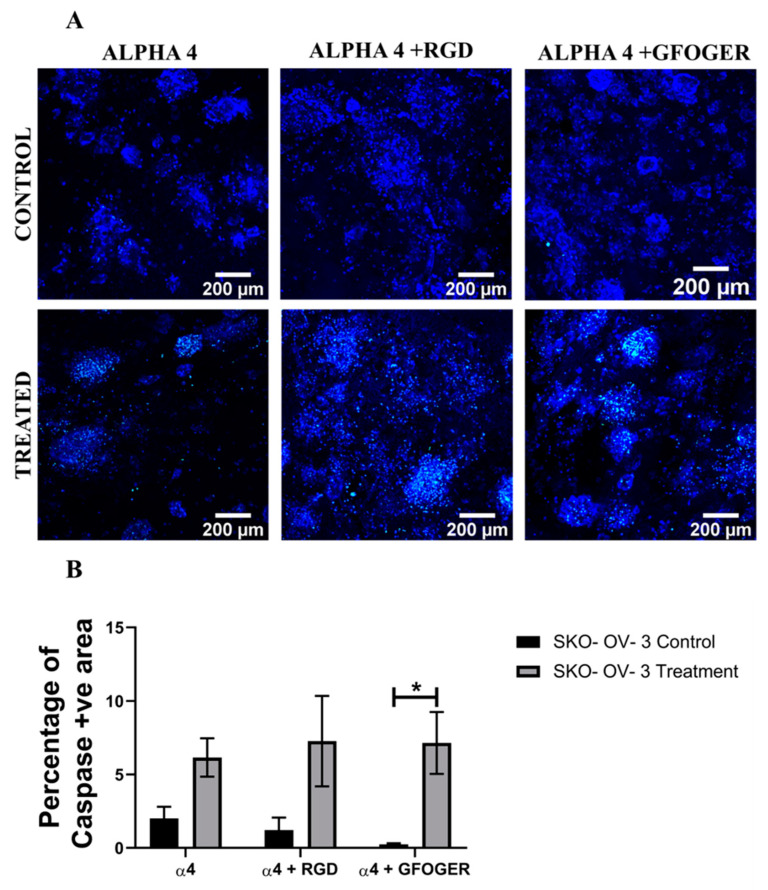
Effect of the chemotherapeutic Cisplatin on the apoptosis of SK-OV-3 EOC cells grown in different PeptiGels, 24 h post-treatment (**A**): Representative images for caspase 3/7 (apoptosis)–DAPI (green–blue) staining for both treated and untreated (control) SK-OV-3 PeptiGels. (**B**) Image analysis-based quantification of apoptotic (green) image areas for SK-OV-3 cells grown in the PeptiGels. Scale bar = 200 µm. Quantitative data represent mean ± SEM for multiple images (≥3) and multiple hydrogels (≥3). * *p* ≤ 0.05.

Figure 3, Figure 4, Figure 5 and Figure 6 show image-based spatial assessment and quantification of the impact of Cisplatin on cell viability and apoptosis induction for A2780 and SK-OV-3 hydrogels of all three configurations. More specifically, Figure 3A and Figure 4A show representative images of live–dead staining for A2780- and SK-OV-3-treated and untreated PeptiGels, respectively, while Figure 3B and Figure 4B show the equivalent quantification (% live areas) from image-based analysis. As observed in Figure 3 and Figure 4, both A 2780 and SK-OV-3 cells were able to attach and proliferate for all three PeptiGel configurations, i.e., α4, α4 + RGD, α4 + GFOGER. However, post Cisplatin treatment, we observed differences in cell viability between the two cell lines. More specifically, a significant loss of cell viability was observed in all three hydrogel configurations for A2780 cells 24 h post-treatment (Figure 3). In contrast, for SK-OV-3, only cells in the α4 hydrogel (without ECM inspired conjugated motifs) showed a significant decrease in cell viability, while SK-OV-3 cells cultured within the biomimetic hydrogels (α4 + RGD and α4 + GFOGER) did not show any significant decrease in cell viability in Cisplatin-treated PeptiGels as compared to untreated controls (Figure 4). 

Analysis of the apoptotic marker caspase 3/7, for both cell lines, in the hydrogels shows a similar trend (Figure 5 and Figure 6). More specifically, for both A2780 and SK-OV-3 cells, an increase in the number of apoptotic cells post Cisplatin treatment was observed in all three hydrogel configurations, but SK-OV-3 had overall less apoptotic cells/less induction of apoptosis in comparison to A2780 for all three hydrogel configurations (Figure 5B and Figure 6B). Taken together, these data suggest that the metastatic cell line SK-OV-3 shows some degree of resistance to Cisplatin treatment in synthetic PeptiGels and that the chemoresistance is substantially increased in the presence of an ECM-inspired peptide conjugation within the hydrogels (Figure 6). In contrast, no such peptide-related Cisplatin resistance is observed for A2780 cells in the PeptiGels (Figure 5).

### 3.3. Assessment of the Impact of the Chemotherapeutic Cisplatin on EOC Cells in an ECM Protein-Coated PU Polymeric Scaffold 3D Model

As previously mentioned, fibronectin- and collagen I-coated polymeric (PU) scaffolds were used for the development and maintenance of long-term (4 weeks) 3D in vitro models of EOC (using A2780 and SK-OV-3 cells). Cisplatin was introduced to the culture medium for both A2780 and SK-OV-3 cell lines 4 weeks into culture at a concentration of 50 µM for 48 h (Section 2.1). Thereafter, similarly to the spheroid (Section 3.1) and PeptiGel (Section 3.2) 3D models, Cisplatin-containing medium was replaced with fresh medium and the polymeric scaffolds were maintained in culture for 24 h, followed by post-treatment analysis of both Cisplatin-treated and untreated A2780 and SK-OV-3 scaffolds of various ECM coatings.

**Figure 7:**
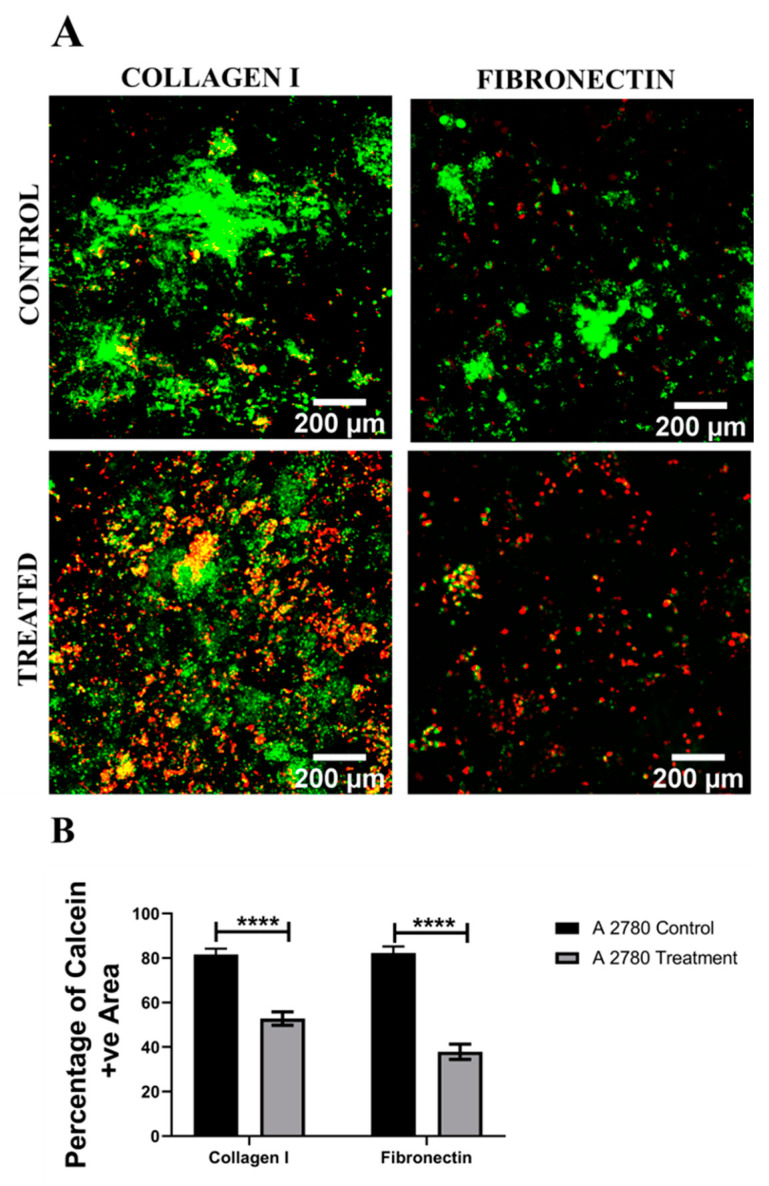
Effect of the chemotherapeutic Cisplatin on the viability of A2780 EOC cells grown in PU scaffolds of various protein coatings, 24 h post-treatment (**A**): Representative images for live–dead (green–red) staining in collagen I- and fibronectin-coated treated and untreated (control) A2780 polymeric scaffolds. (**B**) Image analysis-based quantification of live (green) image areas for A2780 cells grown in collagen I and fibronectin coated polymeric scaffolds. Scale bar = 200 µm. Quantitative data represent mean ± SEM for multiple images (≥3) and multiple scaffolds (≥3). **** *p* ≤ 0.0001.

**Figure 8:**
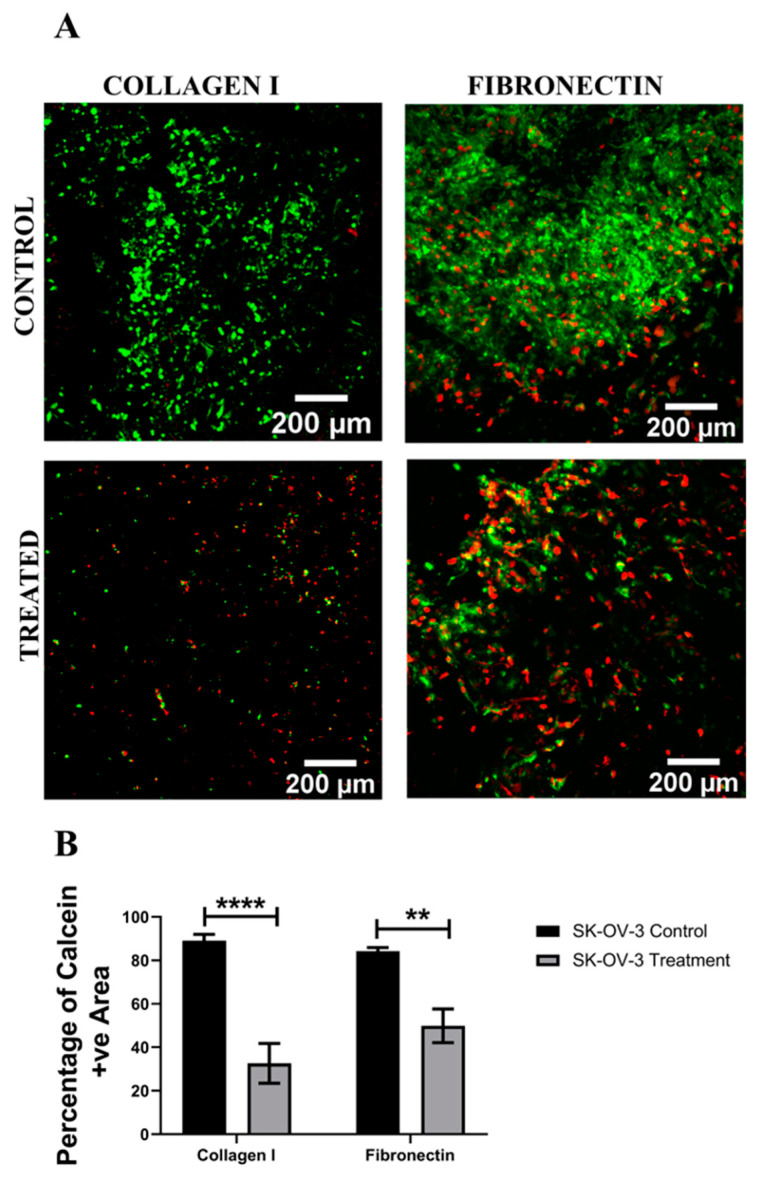
Effect of the chemotherapeutic Cisplatin on the viability of SK-OV-3 EOC cells grown in PU scaffolds of various protein coatings, 24 h post-treatment (**A**): Representative images for live–dead (green–red) staining in collagen I- and fibronectin-coated treated and untreated (control) SK-OV-3 polymeric scaffolds. (**B**) Image analysis-based quantification of live (green) image areas for SK-OV-3 cells grown in collagen I and fibronectin coated polymeric scaffolds. Scale bar = 200 µm. Quantitative data represent mean ± SEM for multiple images (≥3) and multiple scaffolds (≥3). ** *p* ≤ 0.01, **** *p* ≤ 0.0001.

**Figure 9:**
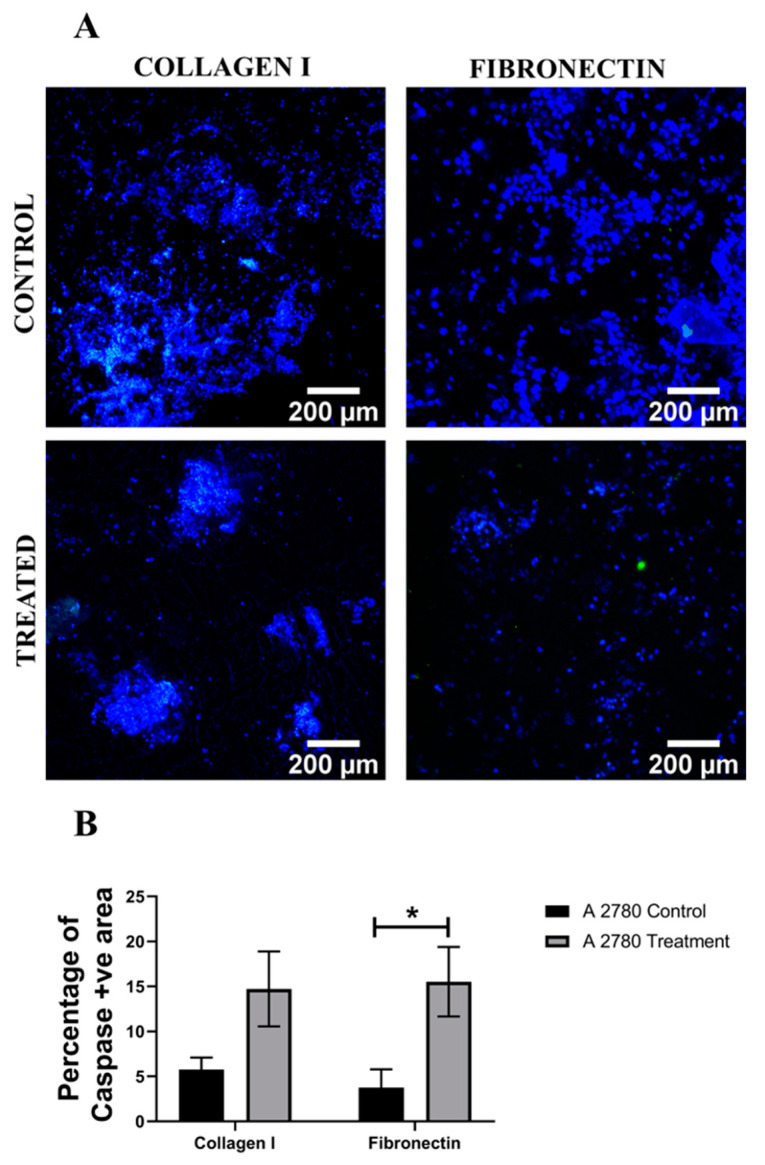
Effect of the chemotherapeutic Cisplatin on the apoptosis A2780 cells grown in PU scaffolds of various protein coatings, 24 h post-treatment (**A**): Representative images for caspase 3/7 (apoptosis)–DAPI (green–blue) staining in collagen I- and fibronectin-coated treated and untreated (control) A2780 polymeric scaffolds (**B**) Image analysis-based quantification of apoptotic (green) image areas for A2780 cells grown in collagen I and fibronectin coated polymeric scaffolds. Scale bar = 200 µm. Quantitative data represent mean ± SEM for multiple images (≥3) and multiple scaffolds (≥3). * *p* ≤ 0.05.

**Figure 10:**
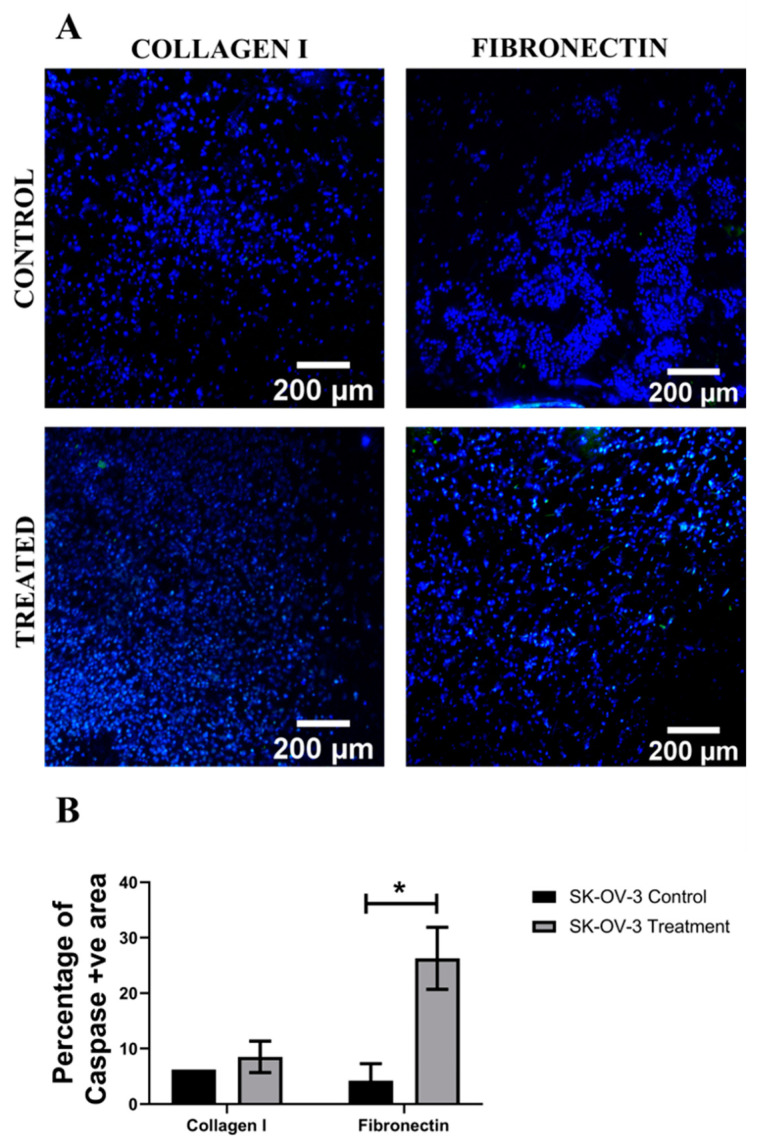
Effect of the chemotherapeutic Cisplatin on the apoptosis of SK-OV-3 cells grown in PU scaffolds of various protein coatings, 24 h post-treatment (**A**): Representative images for caspase 3/7 (apoptosis)–DAPI (green–blue) staining in collagen I- and fibronectin-coated treated and untreated (control) SK-OV-3 polymeric scaffolds. (**B**) Image analysis-based quantification of apoptotic (green) image areas for SK-OV-3 cells grown in collagen I and fibronectin coated polymeric scaffolds. Scale bar = 200 µm. Quantitative data represent mean ± SEM for multiple images (≥3) and multiple scaffolds (≥3). * *p* ≤ 0.05.

Figure 7, Figure 8, Figure 9 and Figure 10 show image-based spatial assessment and quantification of the impact of Cisplatin on the cell viability and apoptosis induction for A2780 and SK-OV-3 cells within fibronectin- and collagen I-coated PU scaffolds. More specifically, Figure 7A and Figure 8A show representative images of live–dead analysis/staining for A2780 and SK-OV-3 scaffolds, respectively. Figure 7B,C and Figure 8B,C show the equivalent quantification of the % of live cell population from image-based analysis on collagen I- and fibronectin-coated scaffolds for A2780 and SK-OV-3, respectively. As observed, PU-based scaffolds were able to maintain a long-term (4 weeks) viable culture for both A2780 and SK-OV-3 cells. Post application of Cisplatin, i.e., 24 h post-treatment, a significant decrease in cell viability was observed for both cell lines, irrespective of the coating (fibronectin or collagen I) of the PU scaffolds (Figure 7B,C and Figure 8B,C). 

Further to cell viability, the induction of cellular apoptosis post Cisplatin treatment in the PU scaffolds was also assessed via caspase 3/7 staining. Figure 9 and Figure 10 show representative images and image-based quantification of apoptosis for Cisplatin-treated and untreated (control) scaffolds for both A2780 and SK-OV-3 cells and both scaffold coatings, i.e., collagen I and fibronectin coating. It was observed that both cell lines showed an increase in the number of apoptotic cells within the scaffolds post Cisplatin treatment. Furthermore, both A2780 and SK-OV-3 showed significantly higher cellular apoptosis in fibronectin-coated scaffolds post-Cisplatin treatment (Figure 9C and Figure 10C). No significant chemoresistance was observed for either of the cell lines within the PU scaffolds, irrespective of the ECM scaffold coating. 

## 4. Discussion

In this work, we have carried out a systematic comparative study to assess the effect of the type of 3D in vitro model/platform on the response of primary and epithelial ovarian cancer cells to the application of chemotherapy (Cisplatin). The 3D models used for this study were (i) simple cell spheroids, (ii) synthetic hydrogels/PeptiGels and (iii) polymeric scaffolds. Two different cell lines were used to assess the effect of the ‘site of cell line origin’, i.e., A2780, which is derived from the ovary (primary site), and SK-OV-3, which is derived from the ascites fluid (metastatic site). Due to the inherent structural differences between the three 3D models, they can be maintained in culture for different time periods. For example, the average time for which spheroids have been maintained in culture is between 6 and 12 days for EOC [35,43,44], while, for hydrogels, it is between 5 and 21 days [53,56,58,61]. There are currently very few studies involving polymeric scaffolds (PLGA–mPEG–PLA, bacterial cellulose–chitosan) and EOC and the average culture duration in these publications was 5–8 days only [63,65,66]. Based on these data, in our systematic study/comparison of different EOC 3D models, we selected different time points for the application of the chemotherapeutic agent Cisplatin. More specifically, Cisplatin was added to spheroids 7 days into culture, while, for hydrogels, it was added 21 days into the culture. Although not with EOC, our group has previously demonstrated that polymeric polyurethane (PU)-based scaffolds are able to support the long-term culture (4–5 weeks) of other cancer cells, including pancreatic cancer and melanoma, along with successful therapeutic assessment [75,76,77,87,88]. Hence, for the PU scaffold-based EOC model, Cisplatin was introduced at the end of 28 days in culture. Currently, there are very few studies where a comparative evaluation of different 3D in vitro models has been carried out for EOC, and they are restricted to comparing either different fabrication methods of the same 3D model, e.g., hanging drop or ultra-low plate for spheroids [74], or different materials such as collagen and alginate for the synthesis of hydrogels [52]. To the best of our knowledge, there are currently no reported studies that compare completely different types of 3D in vitro models for EOC and, with our current study, we are addressing this gap. 

### 4.1. Spheroid EOC Model

As described in Section 2.2 (Methods and Materials), A2780 and SK-OV-3 spheroids were prepared using specialised round-bottom plates and maintained in culture for 7 days, followed by 48 h of Cisplatin (50 µM) treatment and analysis, i.e., viability and apoptosis (live–dead, caspase 3/7) 24 h post-treatment. For both cell lines, we observed morphological differences between untreated controls and Cisplatin-treated spheroids, with the Cisplatin-treated aggregates being less compact than the control spheroids (Figure 1 and Figure 2). This is similar to observations made by Gunay et al. (2020), wherein they reported that the addition of Cisplatin at a concentration of 100 µM disrupted the morphology of EOC spheroids for OVCAR- 3 and OVCAR-8 cell lines [3]. Through live–dead and caspase 3/7 staining, along with image-based quantification (Figure 1 and Figure 2), we observed a cell line-dependent response to the application of Cisplatin within our spheroids. More specifically, A2780 cells that originated from the ovary showed higher cell death 24 h post-chemotherapy in comparison to SK-OV-3 cells, which are ascites-derived (metastatic) (Figure 1). This suggested that SK-OV-3 were more resistant to Cisplatin in comparison to A2780 within our spheroid system. Such a cell line-dependent response for EOC to chemotherapeutic agents including Cisplatin has also been reported by other groups, including Raghavan et al. (2015) and Heredia-Soto et al. (2018) [38,40]. We also observed a certain degree of diffusion limitation within our spheroid models for calcein–ethidium homodimer as well as DAPI–caspase, especially for the compact untreated (control) spheroids (Figure 1A,C and Figure 2A,C). It is well documented that diffusion limitation for nutrients, oxygen and even therapeutic agents is observed in spheroids with diameters higher than 200 µm [89,90,91,92]. Loessner et al. (2010) hypothesise that the phenomenon of cell spheroids displaying elevated chemoresistance to chemotherapeutic agents can be attributed to a number of mechanisms, including decreased penetration of the drugs, increased pro-survival signalling and/or upregulation of genes conferring drug resistance [58]. Although not for EOC, spheroid cultures’ ability to display chemoresistance has also been attributed to the decreased penetration of chemotherapeutic reagents for other cancers (breast, lung and prostrate) by Stock et al. (2016) [92]. Finally, we also observed a high degree of cellular apoptosis for our control spheroids at day 10 of culture (Figure 2A,C), suggesting that the EOC cells within the spheroids had started undergoing programmed cell death, most likely due to a lack of structural integrity and increased diffusional limitations of nutrients and oxygen. This suggests that spheroid models are not suited for long-term culture and that they are more suitable models for rapid therapeutic assessment.

### 4.2. Hydrogel-Based EOC Models

PeptiGels, commercially available synthetic peptide-based materials (Manchester BIOGEL, Manchester, UK) were used to develop our EOC hydrogel models (Section 2.3, Methods and Materials). More specifically, α4 PeptiGel with a stiffness of 1 kPa was used, along with its RGD- and GFOGER-conjugated versions, to compare between a purely synthetic hydrogel and its ECM biomimetic versions, i.e., with RGD mimicking fibronectin and GFOGER mimicking collagen. A2780 and SK-OV-3 EOC cells were grown in all three hydrogel configurations for 21 days, followed by a 48 h Cisplatin (50 µM) treatment and a 24 h post-treatment analysis, i.e., viability, apoptosis (live–dead, caspase 3/7). Both cell lines were able to attach and proliferate and were viable in all three hydrogel configurations for the entire duration of the experiment (Figure 3 and Figure 4). Both cell lines showed a fairly uniform spread and growth within the hydrogels and no aggregates were observed. However, on application of Cisplatin, cellular aggregation within the matrix was observed, which was more pronounced for the A2780 cell line. Similarly to the spheroid model, we again observed a cell line-dependent response to Cisplatin within the hydrogels. More specifically, A2780 showed a significant decrease in cell viability irrespective of the gel type (Figure 3) and a corresponding increase in cellular apoptosis (Figure 5) post-treatment. In contrast, SK-OV-3 showed a significant decrease in cell viability on application of Cisplatin only within the pure α4 synthetic hydrogel. In the presence of RGD and GFOGER motifs, there was very little change in cell viability on Cisplatin application (Figure 4), although an increase in apoptotic cell numbers was observed post-treatment (Figure 6). These data suggest that SK-OV-3 cells show some degree of chemoresistance to Cisplatin within the biomimetic PeptiGels and highlights the importance of ECM proteins in therapeutic resistance for EOC. Although not directly highlighted, as with spheroid models, such cell line-dependent responses to various chemotherapeutic agents for EOC in hydrogel systems have also been reported by other groups in the form of differing IC_50_ [53,61]. For example, Liu et al. (2018) reported that the patient-derived OV-NC cell line had an IC_50_ of 92 ± 3.1 µmol/L in a collagen I gel for carboplatin, while, for the OV-206 patient-derived cell line, it was 154 ± 5.9 for the same drug [61]. They promote two key hypotheses: (i) the chemoresistance observed within the hydrogels can be attributed to the limited delivery of drugs into the core of the tumour model and increased cell survival and (ii) the presence of the collagen matrix can limit the effect of chemotherapy by activating specific signalling pathways, contributing towards chemoresistance via epithelial–mesenchymal transition (EMT) [61]. These hypotheses justify our observations within the PeptiGels and highlight the role of ECM proteins in chemoresistance for EOC. The role of the RGD motif in conferring chemoresistance to EOC has also been reported by Bondong et al. (2012) in their analysis of an ovarian cancer patient tumour and ascites fluid. They reported that the overexpression of the L1 cell adhesion molecule (L1CAM) is linked to poor prognosis in patients. Specifically, L1CAM, present in the ascites fluid, contains an RGD motif and is linked to the development of chemoresistance of EOC amongst patients [93]. Their observation is in line with our data where we see increased chemoresistance for the ascites-derived cell line SK-OV-3 (metastatic) within RGD-conjugated PeptiGels (Figure 4). Similarly, the role of collagen in the chemoresistance of EOC has also been reported by some other researchers [84,94]. For example, Januchowski et al. (2016) reported the overexpression of a number of different types of collagen (COL1A1, COL5A2, COL1A2, COL15A1, COL3A1, etc.) in chemotherapy-resistant versions of different EOC cell lines. including A2780 and SK-OV-3 [84]. They attribute this to cell adhesion-mediated drug resistance (CAM-DR) and suggest that the interaction of ECM components, including collagen, with the cancer cells results in chemoresistance. These interactions can even change the apoptosis sensitivity and increase the drug resistance of cancer cells [85,86]. Our observation of chemoresistance for SK-OV-3 (Figure 4 and Figure 6) in RGD- and collagen I (GFOGER)-conjugated PeptiGels could also be due to the CAM-DR phenomenon, although further studies are needed to validate this. 

### 4.3. Polymeric Scaffold-Based EOC Models

We have previously developed a highly porous and biocompatible PU scaffold via the TIPS method and have shown that a number of different cell types can be cultured long-term within these scaffolds [75,76,77,87]. Based on the timeframe of cell culture in the polymeric scaffolds reported in our previous publications, EOC cell lines in this study were grown within the polymeric scaffolds for 28 days, followed by 48 h of Cisplatin (50 µM) treatment and a 24 h post-treatment analysis, i.e., for viability and apoptosis. Collagen I- and fibronectin-coated scaffolds were used in line with our previous observations on the importance of ECM mimicry within these scaffolds for both healthy and diseased (cancerous) cells [76,77]. The choice of collagen I and fibronectin also allowed us to compare between the ECM protein-coated scaffolds and the peptide-conjugated PeptiGels to some extent. As observed in Figure 7 and Figure 8, both A2780 and SK-OV-3 cell lines were able to grow for 28 days within our polymeric scaffolds, irrespective of the coating protein. On application of Cisplatin, both cell lines showed a significant drop in cell viability within the scaffolds, irrespective of the ECM protein present (Figure 7 and Figure 8). Similarly, the number of apoptotic cells also increased post-treatment within the scaffolds for both cell lines (Figure 9 and Figure 10). For A2780, the cell response to Cisplatin within the PU scaffolds was similar to that observed within our spheroids and hydrogel models (Figure 1, Figure 2 and Figure 3 and Figure 5) However, our results with SK-OV-3 in the scaffolds contradict our observations within the spheroid and hydrogel models, wherein the SK-OV-3 cell line showed some degree of Cisplatin resistance, which was particularly pronounced in the presence of ECM protein mimicry (RGD, GFOGER) within the hydrogel models (Figure 3 and Figure 4). To the best of our knowledge, there is currently no available literature for the chemotherapeutic assessment of ovarian cancer within polymeric scaffolds to enable us to compare our data. It is possible that the relatively large pore size (100–150 µm) and highly interconnected pores of the PU scaffolds allowed for the extensive diffusion of Cisplatin, which affected A2780 and SK-OV-3′s response to chemotherapy. This is a feasible theory since diffusion limitation of therapeutic agents within spheroids and hydrogels has been considered to be one of the reasons for the chemoresistance of EOC cells observed within these systems [58,61]. Another reason for this difference in SK-OV-3′s response to Cisplatin can be the difference in stiffness between the models. The elastic modulus for our PU scaffolds is around 28 ± 3 kPa [77,95], while the elastic modulus of α4 hydrogel is only 1 kPa. SK-OV-3′s preferential chemoresistance on softer matrices has been reported by other groups too [96,97]. For example, Fan et al. (2021) cultured SK-OV-3 cell lines on glass sheets coated with hydrogel substrates of varying stiffness (0.5–25 kPa). They observed that SK-OV-3 showed higher chemoresistance to Cisplatin and Paclitaxel on softer substrates and linked it to the overexpression of ABC transporters ABCB1 and ABCB4 on the soft substrates, which are genes linked to the development of multidrug resistance [96]. McGrail et al. (2014) carried out a comparative study between SK-OV-3 (highly metastatic) and OVCAR-3 (less metastatic) cell lines on Polyacrylamide substrates coated with equal densities of collagen I with two different stiffness types, i.e., 2.83 kPa mimicking adipocytes and 34.88 kPa mimicking osteoblasts [97]. They reported that the SK-OV-3 cell line was more mechanosensitive than OVCAR-3, resulting in a display of higher malignancy, a mesenchymal phenotype and higher resistance to Carboplatin on a softer substrate [97]. The findings of these studies are in line with our observation of chemoresistance by the SK-OV-3 cell line on the softer spheroid and hydrogel models as compared to the stiffer PU polymeric scaffold model.

Overall, in this work, we have conducted a novel, systematic comparative study of the response of EOC to a chemotherapeutic (Cisplatin) in three different in vitro 3D models: (i) spheroids, (ii) synthetic PeptiGels/hydrogels and (iii) polymeric scaffolds with various ECM coatings. Two different cell lines (A2780 and SK-OV-3) were used to understand the impact of the ‘site of origin’ of the cells, as well as to assess platform versatility. We have reported that all three platforms were able to support EOC in vitro 3D models with both cell lines, albeit for different culture time points. Polymeric scaffolds and hydrogels were maintained for 4 weeks and 3 weeks, respectively (Figure 3, Figure 4, Figure 7 and Figure 8), highlighting that they are suitable models for the long-term 3D culture of EOC cell lines. Spheroids were able to survive for a shorter time period of around 7 days (Figure 1). On application of Cisplatin, A2780 cells (primary) showed a decrease in viable cell population across all three platforms (Figure 1, Figure 3 and Figure 7). In comparison, SK-OV-3 cells (metastatic) showed decreased cell viability on chemotherapeutic application only on the PU polymeric scaffold (Figure 8) but showed resistance to Cisplatin when grown as spheroids (Figure 1) or within hydrogels, with the latter being peptide conjugation-dependent (Figure 4). More specifically, SK-OV-3′s Cisplatin resistance within hydrogels was observed in the presence of conjugated ECM protein motifs (RGD and GFOGER), highlighting the importance of ECM proteins in the chemoresistance of metastatic EOC. Our data also show that the response to chemotherapy is dependent on the cell site/location of origin of the cells. To date, most comparative studies for different 3D models have usually used the same platform system and focused on either different materials or on different fabrication methods of the models. To the best of our knowledge, this is the first time that such a comparative study for three completely different 3D in vitro models, across different culture time points and with different cell lines, has been carried out.

## 5. Conclusions

The goal of the present study was to carry out, for the first time, a robust comparative study of the growth and chemotherapy response of EOC in three widely different in vitro 3D models: (i) cell spheroids, (ii) synthetic PeptiGels/hydrogels and (iii) polymeric scaffolds of various protein coatings. We have shown the feasibility of using all three models for the culture of EOC cell lines A2780 and SK-OV-3, representing primary and metastatic disease, respectively, and assessed the impact of chemotherapy (Cisplatin) on the cell viability and apoptosis within these models. Our study highlights that the selection of a 3D in vitro platform depends on (i) the planned experimental/assessment time period, (ii) the type of cell to be studied, (iii) the site of cell origin in vivo and (iv) the question that needs to be answered. For example, a rapid screening analysis may benefit from the use of simple cell spheroid models; however, the need to study the effect of ECM proteins on cell growth and chemotherapy response long-term will benefit from structured hydrogel or polymeric scaffolds. Similarly, softer tumours or tumours originating from soft tissues such as the ovary or the omentum may prefer less stiff 3D platforms such as spheroids or hydrogels as compared to stiffer polymeric scaffolds. In conclusion, our study highlights that, as with most tissue engineering applications, there is no ‘one-size-fits-all model’ [92] and the selection of an appropriate model requires careful assessment of the available input variables and the expected outputs.

## Data Availability

The data presented in this study are available on request from the corresponding author.

## References

[1] Augustine R., Kalva S.N., Ahmad R., Zahid A.A., Hasan S., Nayeem A., McClements L., Hasan A. (2021). 3D Bioprinted cancer models: Revolutionizing personalized cancer therapy. Transl. Oncol..

[2] Trinidad C.V., Tetlow A.L., Bantis L.E., Godwin A.K. (2020). Reducing Ovarian Cancer Mortality Through Early Detection: Approaches Using Circulating Biomarkers. Cancer Prev. Res..

[3] Ahmed N., Kadife E., Raza A., Short M., Jubinsky P.T., Kannourakis G. (2020). Ovarian Cancer, Cancer Stem Cells and Current Treatment Strategies: A Potential Role of Magmas in the Current Treatment Methods. Cells.

[4] Nowacka M., Sterzynska K., Andrzejewska M., Nowicki M., Januchowski R. (2021). Drug resistance evaluation in novel 3D in vitro model. Biomed. Pharmacother..

[5] Peña C.M., Skipper T., Hsu J., Schechter I., Ghosh D., Dawson M. (2021). Development of a Novel 3D Model to Investigate the Role of Heterogeneity in Ovarian Cancer Chemoresistance. FASEB J..

[6] Momenimovahed Z., Tiznobaik A., Taheri S., Salehiniya H. (2019). Ovarian cancer in the world: Epidemiology and risk factors. Int. J. Women’s Health.

[7] Rosen B., Laframboise S., Ferguson S., Dodge J., Bernardini M., Murphy J., Segev Y., Sun P., Narod S.A. (2014). The impacts of neoadjuvant chemotherapy and of debulking surgery on survival from advanced ovarian cancer. Gynecol. Oncol..

[8] Redman C.W.E., Warwick J., Luesley D.M., Varma R., Lawton F.G., Blackledge G.R.P. (1994). Intervention debulking surgery in advanced epithelial ovarian cancer. BJOG Int. J. Obstet. Gynaecol..

[9] Vergote I., Amant F., Kristensen G., Ehlen T., Reed N.S., Casado A. (2011). Primary surgery or neoadjuvant chemotherapy followed by interval debulking surgery in advanced ovarian cancer. Eur. J. Cancer.

[10] Board P.A.T.E. (2021). Ovarian Epithelial, Fallopian Tube, and Primary Peritoneal Cancer Treatment (PDQ®): Patient Version. PDQ Cancer Information Summaries.

[11] Baci D., Bosi A., Gallazzi M., Rizzi M., Noonan D.M., Poggi A., Bruno A., Mortara L. (2020). The Ovarian Cancer Tumor Immune Microenvironment (TIME) as Target for Therapy: A Focus on Innate Immunity Cells as Therapeutic Effectors. Int. J. Mol. Sci..

[12] Westergaard M.C.W., Milne K., Pedersen M., Hasselager T., Olsen L.R., Anglesio M.S., Borch T.H., Kennedy M., Briggs G., LeDoux S. (2020). Changes in the Tumor Immune Microenvironment during Disease Progression in Patients with Ovarian Cancer. Cancers.

[13] Jiang Y., Wang C., Zhou S. (2020). Targeting tumor microenvironment in ovarian cancer: Premise and promise. Biochim. Biophys. Acta.

[14] Beaufort C.M., Helmijr J.C.A., Piskorz A.M., Hoogstraat M., Ruigrok-Ritstier K., Besselink N., Murtaza M., van Ijcken W., Heine A., Smid M. (2014). Ovarian Cancer Cell Line Panel (OCCP): Clinical Importance of In Vitro Morphological Subtypes. PLoS ONE.

[15] Buick R.N., Pullano R., Trent J.M. (1985). Comparative properties of five human ovarian adenocarcinoma cell lines. Cancer Res..

[16] Haley J., Tomar S., Pulliam N., Xiong S., Perkins S.M., Karpf A.R., Mitra S., Nephew K.P., Mitra A.K. (2016). Functional characterization of a panel of high-grade serous ovarian cancer cell lines as representative experimental models of the disease. Oncotarget.

[17] Hernandez L., Kim M.K., Lyle L.T., Bunch K.P., House C.D., Ning F., Noonan A.M., Annunziata C.M. (2016). Characterization of ovarian cancer cell lines as in vivo models for preclinical studies. Gynecol. Oncol..

[18] Havrilesky L.J., Elbendary A., Hurteau J.A., Whitaker R.S., Rodriguez G.C., Berchuck A.W. (1995). Chemotherapy-induced apoptosis in epithelial ovarian cancers. Obstet. Gynecol..

[19] Johnson P.A., Giles J.R. (2013). The hen as a model of ovarian cancer. Nat. Cancer.

[20] Roby K.F., Taylor C.C., Sweetwood J.P., Cheng Y., Pace J.L., Tawfik O., Persons D.L., Smith P., Terranova P.F. (2000). Development of a syngeneic mouse model for events related to ovarian cancer. Carcinogenesis.

[21] Stakleff K.S., Rouse A., Ryan A., Haller N., Von Gruenigen V. (2005). A novel early-stage orthotopic model for ovarian cancer in the Fischer 344 rat. Int. J. Gynecol. Cancer.

[22] Brodeur M.N., Simeone K., Leclerc-Deslauniers K., Fleury H., Carmona E., Provencher D.M., Mes-Masson A.-M. (2021). Carboplatin response in preclinical models for ovarian cancer: Comparison of 2D monolayers, spheroids, ex vivo tumors and in vivo models. Sci. Rep..

[23] Hadi L.M., Yaghini E., MacRobert A.J., Loizidou M. (2020). Synergy between Photodynamic Therapy and Dactinomycin Chemotherapy in 2D and 3D Ovarian Cancer Cell Cultures. Int. J. Mol. Sci..

[24] Sonoda T., Kobayashi H., Kaku T., Hirakawa T., Nakano H. (2003). Expression of angiogenesis factors in monolayer culture, multicellular spheroid and in vivo transplanted tumor by human ovarian cancer cell lines. Cancer Lett..

[25] Chim L.K., Mikos A.G. (2018). Biomechanical forces in tissue engineered tumor models. Curr. Opin. Biomed. Eng..

[26] Totti S., Vernardis S., Meira L., Pérez-Mancera P.A., Costello E., Greenhalf W., Palmer D., Neoptolemos J., Mantalaris A., Velliou E.G. (2017). Designing a bio-inspired biomimetic in vitro system for the optimization of ex vivo studies of pancreatic cancer. Drug Discov. Today.

[27] Goff B., Blake J., Bamberg M., Hasan T. (1996). Treatment of ovarian cancer with photodynamic therapy and immunoconjugates in a murine ovarian cancer model. Br. J. Cancer.

[28] Konstantinopoulos P.A., Matulonis U.A. (2013). Current Status and Evolution of Preclinical Drug Development Models of Epithelial Ovarian Cancer. Front. Oncol..

[29] Magnotti E., Marasco W.A. (2018). The latest animal models of ovarian cancer for novel drug discovery. Expert Opin. Drug Discov..

[30] McCloskey C.W., Rodriguez G.M., Galpin K.J.C., Vanderhyden B.C. (2018). Ovarian Cancer Immunotherapy: Preclinical Models and Emerging Therapeutics. Cancers.

[31] Erstad D.J., Sojoodi M., Taylor M., Ghoshal S., Razavi A.A., Graham-O’Regan K.A., Bardeesy N., Ferrone C.R., Lanuti M., Caravan P. (2018). Orthotopic and heterotopic murine models of pancreatic cancer and their different responses to FOLFIRINOX chemotherapy. Dis. Model. Mech..

[32] Nyga A., Cheema U., Loizidou M. (2011). 3D tumour models: Novel in vitro approaches to cancer studies. J. Cell Commun. Signal..

[33] Tentler J.J., Tan A.C., Weekes C.D., Jimeno A., Leong S., Pitts T.M., Arcaroli J.J., Messersmith W.A., Eckhardt S.G. (2012). Patient-derived tumour xenografts as models for oncology drug development. Nat. Rev. Clin. Oncol..

[34] Johnson J., Decker S., Zaharevitz D., Rubinstein L.V., Venditti J.M., Schepartz S., Kalyandrug S., Christian M., Arbuck S., Hollingshead M. (2001). Relationships between drug activity in NCI preclinical in vitro and in vivo models and early clinical trials. Br. J. Cancer.

[35] Raghavan S., Mehta P., Ward M.R., Bregenzer M.E., Fleck E.M.A., Tan L., McLean K., Buckanovich R.J., Mehta G. (2017). Personalized Medicine–Based Approach to Model Patterns of Chemoresistance and Tumor Recurrence Using Ovarian Cancer Stem Cell Spheroids. Clin. Cancer Res..

[36] Liao J., Qian F., Tchabo N., Mhawech-Fauceglia P., Beck A., Qian Z., Wang X., Huss W.J., Lele S.B., Morrison C.D. (2014). Ovarian Cancer Spheroid Cells with Stem Cell-Like Properties Contribute to Tumor Generation, Metastasis and Chemotherapy Resistance through Hypoxia-Resistant Metabolism. PLoS ONE.

[37] Lal-Nag M., McGee L., Titus S.A., Brimacombe K., Michael S., Sittampalam G., Ferrer M. (2017). Exploring Drug Dosing Regimens In Vitro Using Real-Time 3D Spheroid Tumor Growth Assays. SLAS Discov. Adv. Sci. Drug Discov..

[38] Heredia-Soto V., Redondo A., Berjón A., Miguel-Martín M., Díaz E., Crespo R., Hernández A., Yébenes L., Gallego A., Feliu J. (2018). High-throughput 3-dimensional culture of epithelial ovarian cancer cells as preclinical model of disease. Oncotarget.

[39] Shuford S., Wilhelm C., Rayner M., Elrod A., Millard M., Mattingly C., Lotstein A., Smith A.M., Guo Q.J., O’Donnell L. (2019). Prospective Validation of an Ex Vivo, Patient-Derived 3D Spheroid Model for Response Predictions in Newly Diagnosed Ovarian Cancer. Sci. Rep..

[40] Raghavan S., Ward M.R., Rowley K.R., Wold R.M., Takayama S., Buckanovich R.J., Mehta G. (2015). Formation of stable small cell number three-dimensional ovarian cancer spheroids using hanging drop arrays for preclinical drug sensitivity assays. Gynecol. Oncol..

[41] Rafehi S., Valdes Y.R., Bertrand M., McGee J., Préfontaine M., Sugimoto A., DiMattia G., Shepherd T.G. (2015). TGFβ signaling regulates epithelial–mesenchymal plasticity in ovarian cancer ascites-derived spheroids. Endocr.-Relat. Cancer.

[42] Puiffe M.-L., Le Page C., Filali-Mouhim A., Zietarska M., Ouellet V., Tonin P.N., Chevrette M., Provencher D.M., Mes-Masson A.-M. (2007). Characterization of Ovarian Cancer Ascites on Cell Invasion, Proliferation, Spheroid Formation, Gene Expression in an In Vitro Model of Epithelial Ovarian Cancer. Neoplasia.

[43] Griffon G., Marchal C., Merlin J.-L., Parache R., Bey P. (1995). Radiosensitivity of multicellular tumour spheroids obtained from human ovarian cancers. Eur. J. Cancer.

[44] Gunay G., Kirit H.A., Kamatar A., Baghdasaryan O., Hamsici S., Acar H. (2020). The effects of size and shape of the ovarian cancer spheroids on the drug resistance and migration. Gynecol. Oncol..

[45] Zhang S., Balch C., Chan M., Lai H.-C., Matei D., Schilder J.M., Yan P.S., Huang T.H.-M., Nephew K.P. (2008). Identification and Characterization of Ovarian Cancer-Initiating Cells from Primary Human Tumors. Cancer Res..

[46] Shield K., Ackland L., Ahmed N., Rice G. (2009). Multicellular spheroids in ovarian cancer metastases: Biology and pathology. Gynecol. Oncol..

[47] Masiello T., Dhall A., Hemachandra L.P.M., Tokranova N., Melendez J.A., Castracane J. (2018). A Dynamic Culture Method to Produce Ovarian Cancer Spheroids under Physiologically-Relevant Shear Stress. Cells.

[48] Li S.-S., Ip C.K., Tang M.Y.H., Sy S.K.H., Yung S., Chan T.-M., Yang M., Shum H.C., Wong A.S. (2017). Modeling Ovarian Cancer Multicellular Spheroid Behavior in a Dynamic 3D Peritoneal Microdevice. J. Vis. Exp..

[49] Lawrenson K., Mhawech-Fauceglia P., Worthington J., Spindler T.J., O’Brien D., Lee J.M., Spain G., Sharifian M., Wang G., Darcy K.M. (2015). Identification of novel candidate biomarkers of epithelial ovarian cancer by profiling the secretomes of three-dimensional genetic models of ovarian carcinogenesis. Int. J. Cancer.

[50] Kapałczyńska M., Kolenda T., Przybyła W., Zajączkowska M., Teresiak A., Filas V., Ibbs M., Bliźniak R., Łuczewski L., Lamperska K. (2018). 2D and 3D cell cultures—A comparison of different types of cancer cell cultures. Arch. Med. Sci..

[51] Fang Y., Eglen R.M. (2017). Three-Dimensional Cell Cultures in Drug Discovery and Development. SLAS Discov. Adv. Sci. Drug Discov..

[52] Zheng L., Hu X., Huang Y., Xu G., Yang J., Li L. (2015). In vivo bioengineered ovarian tumors based on collagen, matrigel, alginate and agarose hydrogels: A comparative study. Biomed. Mater..

[53] Yang Z., Zhao X. (2011). A 3D model of ovarian cancer cell lines on peptide nanofiber scaffold to explore the cell–scaffold interaction and chemotherapeutic resistance of anticancer drugs. Int. J. Nanomed..

[54] Xu G., Yin F., Wu H., Hu X., Zheng L., Zhao J. (2014). In vitro ovarian cancer model based on three-dimensional agarose hydrogel. J. Tissue Eng..

[55] Xu F., Celli J., Rizvi I., Moon S., Hasan T., Demirci U. (2011). A three-dimensional in vitro ovarian cancer coculture model using a high-throughput cell patterning platform. Biotechnol. J..

[56] Hedegaard C.L., Redondo-Gómez C., Tan B.Y., Ng K.W., Loessner D., Mata A. (2020). Peptide-protein coassembling matrices as a biomimetic 3D model of ovarian cancer. Sci. Adv..

[57] Sodek K.L., Brown T.J., Ringuette M.J. (2008). Collagen I but not Matrigel matrices provide an MMP-dependent barrier to ovarian cancer cell penetration. BMC Cancer.

[58] Loessner D., Stok K.S., Lutolf M.P., Hutmacher D.W., Clements J.A., Rizzi S.C. (2010). Bioengineered 3D platform to explore cell–ECM interactions and drug resistance of epithelial ovarian cancer cells. Biomaterials.

[59] Chen J., Wang J., Zhang Y., Chen D., Yang C., Kai C., Wang X., Shi F., Dou J. (2014). Observation of ovarian cancer stem cell behavior and investigation of potential mechanisms of drug resistance in three-dimensional cell culture. J. Biosci. Bioeng..

[60] Lachowski D., Matellan C., Cortes E., Saiani A., Miller A., Hernández A.D.R. (2021). Self-Assembling Polypeptide Hydrogels as a Platform to Recapitulate the Tumor Microenvironment. Cancers.

[61] Liu M., Zhang X., Long C., Xu H., Cheng X., Chang J., Zhang C., Zhang C., Wang X. (2018). Collagen-based three-dimensional culture microenvironment promotes epithelial to mesenchymal transition and drug resistance of human ovarian cancer in vitro. RSC Adv..

[62] Wan X., Ball S., Willenbrock F., Yeh S., Vlahov N., Koennig D., Green M., Brown G., Jeyaretna D., Delia K. (2017). Perfused Three-dimensional Organotypic Culture of Human Cancer Cells for Therapeutic Evaluation. Sci. Rep..

[63] Girard Y.K., Wang C., Ravi S., Howell M.C., Mallela J., Alibrahim M., Green R., Hellermann G., Mohapatra S.S., Mohapatra S. (2013). A 3D Fibrous Scaffold Inducing Tumoroids: A Platform for Anticancer Drug Development. PLoS ONE.

[64] Alkmin S., Brodziski R., Simon H., Hinton D., Goldsmith R.H., Patankar M., Campagnola P.J. (2020). Role of Collagen Fiber Morphology on Ovarian Cancer Cell Migration Using Image-Based Models of the Extracellular Matrix. Cancers.

[65] Ul-Islam M., Subhan F., Islam S.U., Khan S., Shah N., Manan S., Ullah M.W., Yang G. (2019). Development of three-dimensional bacterial cellulose/chitosan scaffolds: Analysis of cell-scaffold interaction for potential application in the diagnosis of ovarian cancer. Int. J. Biol. Macromol..

[66] De Jaeghere E.A., De Vlieghere E., Van Hoorick J., Van Vlierberghe S., Wagemans G., Pieters L., Melsens E., Praet M., Van Dorpe J., Boone M.N. (2018). Heterocellular 3D scaffolds as biomimetic to recapitulate the tumor microenvironment of peritoneal metastases in vitro and in vivo. Biomaterials.

[67] Avraham-Chakim L., Elad D., Zaretsky U., Kloog Y., Jaffa A., Grisaru D. (2013). Fluid-Flow Induced Wall Shear Stress and Epithelial Ovarian Cancer Peritoneal Spreading. PLoS ONE.

[68] Matte I., Legault C.M., Garde-Granger P., Laplante C., Bessette P., Rancourt C., Piché A. (2016). Mesothelial cells interact with tumor cells for the formation of ovarian cancer multicellular spheroids in peritoneal effusions. Clin. Exp. Metastasis.

[69] Kenny H.A., Krausz T., Yamada S.D., Lengyel E. (2007). Use of a novel 3D culture model to elucidate the role of mesothelial cells, fibroblasts and extra-cellular matrices on adhesion and invasion of ovarian cancer cells to the omentum. Int. J. Cancer.

[70] Ip C.K., Li S.-S., Tang M.Y.H., Sy S.K.H., Ren Y., Shum H.C., Wong A.S.T. (2016). Stemness and chemoresistance in epithelial ovarian carcinoma cells under shear stress. Sci. Rep..

[71] Hyler A.R., Baudoin N.C., Brown M.S., Stremler M., Cimini D., Davalos R.V., Schmelz E.M. (2018). Fluid shear stress impacts ovarian cancer cell viability, subcellular organization, and promotes genomic instability. PLoS ONE.

[72] Zhang Y., Tang H., Cai J., Zhang T., Guo J., Feng D., Wang Z. (2011). Ovarian cancer-associated fibroblasts contribute to epithelial ovarian carcinoma metastasis by promoting angiogenesis, lymphangiogenesis and tumor cell invasion. Cancer Lett..

[73] Brooks E.A., Gencoglu M.F., Corbett D.C., Stevens K.R., Peyton S.R. (2019). An omentum-inspired 3D PEG hydrogel for identifying ECM-drivers of drug resistant ovarian cancer. APL Bioeng..

[74] Patra B., Lateef M.A., Brodeur M.N., Fleury H., Carmona E., Péant B., Provencher D., Mes-Masson A.-M., Gervais T. (2020). Carboplatin sensitivity in epithelial ovarian cancer cell lines: The impact of model systems. PLoS ONE.

[75] Gupta P., Totti S., Pérez-Mancera P.A., Dyke E., Nisbet A., Schettino G., Webb R., Velliou E.G. (2019). Chemoradiotherapy screening in a novel biomimetic polymer based pancreatic cancer model. RSC Adv..

[76] Gupta P., Pérez-Mancera P.A., Kocher H., Nisbet A., Schettino G., Velliou E.G. (2020). A Novel Scaffold-Based Hybrid Multicellular Model for Pancreatic Ductal Adenocarcinoma—Toward a Better Mimicry of the in vivo Tumor Microenvironment. Front. Bioeng. Biotechnol..

[77] Totti S., Allenby M.C., Dos Santos S.B., Mantalaris A., Velliou E.G. (2018). A 3D bioinspired highly porous polymeric scaffolding system for in vitro simulation of pancreatic ductal adenocarcinoma. RSC Adv..

[78] Echo A., Howell V.M., Colvin E.K. (2015). The Extracellular Matrix in Epithelial Ovarian Cancer—A Piece of a Puzzle. Front. Oncol..

[79] Kenny H.A., Lengyel E. (2009). MMP-2 functions as an early response protein in ovarian cancer metastasis. Cell Cycle.

[80] Kumar D., Workman V., O’Brien M., McLaren J., White L., Ragunath K., Rose F., Saiani A., Gough J.E. (2017). Peptide Hydrogels-A Tissue Engineering Strategy for the Prevention of Oesophageal Strictures. Adv. Funct. Mater..

[81] Allenby M.C., Misener R., Panoskaltsis N., Mantalaris A. (2017). A Quantitative Three-Dimensional Image Analysis Tool for Maximal Acquisition of Spatial Heterogeneity Data. Tissue Eng. Part C Methods.

[82] Allenby M.C., Panoskaltsis N., Tahlawi A., Dos Santos S.B., Mantalaris A. (2018). Dynamic human erythropoiesis in a three-dimensional perfusion bone marrow biomimicry. Biomaterials.

[83] Tahlawi A., Klontzas M.E., Allenby M.C., Morais J.C., Panoskaltsis N., Mantalaris A. (2018). RGD-functionalized polyurethane scaffolds promote umbilical cord blood mesenchymal stem cell expansion and osteogenic differentiation. J. Tissue Eng. Regen. Med..

[84] Januchowski R., Świerczewska M., Sterzyńska K., Wojtowicz K., Nowicki M., Zabel M. (2016). Increased Expression of Several Collagen Genes is Associated with Drug Resistance in Ovarian Cancer Cell Lines. J. Cancer.

[85] Morin P.J. (2003). Drug resistance and the microenvironment: Nature and nurture. Drug Resist. Updat..

[86] Croix B.S., Kerbel R.S. (1997). Cell adhesion and drug resistance in cancer. Curr. Opin. Oncol..

[87] Totti S., Ng K.W., Dale L., Lian G., Chen T., Velliou E.G. (2019). A novel versatile animal-free 3D tool for rapid low-cost assessment of immunodiagnostic microneedles. Sens. Actuators B Chem..

[88] Wishart G., Gupta P., Nisbet A., Schettino G., Velliou E. (2021). On the Evaluation of a Novel Hypoxic 3D Pancreatic Cancer Model as a Tool for Radiotherapy Treatment Screening. Cancers.

[89] Frankel A., Buckman R., Kerbel R.S. (1997). Abrogation of taxol-induced G2-M arrest and apoptosis in human ovarian cancer cells grown as multicellular tumor spheroids. Cancer Res..

[90] Mehta G., Hsiao A.Y., Ingram M., Luker G.D., Takayama S. (2012). Opportunities and challenges for use of tumor spheroids as models to test drug delivery and efficacy. J. Control Release.

[91] Folkman J., Hochberg M. (1973). SELF-REGULATION OF GROWTH IN THREE DIMENSIONS. J. Exp. Med..

[92] Stock K., Estrada M., Vidic S., Gjerde K., Rudisch A., Santo V.E., Barbier M., Blom S., Arundkar S.C., Selvam I. (2016). Capturing tumor complexity in vitro: Comparative analysis of 2D and 3D tumor models for drug discovery. Sci. Rep..

[93] Bondong S., Kiefel H., Hielscher T., Zeimet A.G., Zeillinger R., Pils D., Schuster E., Castillo-Tong D.C., Cadron I., Vergote I. (2012). Prognostic significance of L1CAM in ovarian cancer and its role in constitutive NF-κB activation. Ann. Oncol..

[94] Sterzyńska K., Klejewski A., Wojtowicz K., Świerczewska M., Nowacka M., Kaźmierczak D., Andrzejewska M., Rusek D., Brązert M., Brązert J. (2018). Mutual Expression of ALDH1A1, LOX, and Collagens in Ovarian Cancer Cell Lines as Combined CSCs- and ECM-Related Models of Drug Resistance Development. Int. J. Mol. Sci..

[95] Safinia L., Mantalaris A., Bismarck A. (2006). Nondestructive Technique for the Characterization of the Pore Size Distribution of Soft Porous Constructs for Tissue Engineering. Langmuir.

[96] Fan Y., Sun Q., Li X., Feng J., Ao Z., Li X., Wang J. (2021). Substrate Stiffness Modulates the Growth, Phenotype, and Chemoresistance of Ovarian Cancer Cells. Front. Cell Dev. Biol..

[97] McGrail D.J., Kieu Q.M.N., Dawson M.R. (2014). The malignancy of metastatic ovarian cancer cells is increased on soft matrices through a mechanosensitive Rho–ROCK pathway. J. Cell Sci..

